# Recognition Mechanism of Dangerous Goods Marks: Evidence from an Event-Related Potential Study

**DOI:** 10.3390/ijerph20065192

**Published:** 2023-03-15

**Authors:** Qiang Wei, Xinyu Du, Yixin Lin, Guanhua Hou, Siyuan Liu, Hao Fang, Ming Jin

**Affiliations:** 1 School of Education, Jianghan University, Wuhan 430056, China; 2 School of Arts and Communication, China University of Geoscience, Wuhan 430074, China; 3 School of Energy and Power Engineering, Huazhong University of Science and Technology, Wuhan 430074, China; 4 Pan Tianshou College of Architecture, Art and Design, Ningbo University, Ningbo 315211, China; 5 School of Art and Design, Wuhan Institute of Technology, Wuhan 430205, China; 6 Engineering Research Center of Big Data Application in Private Health Medicine, Fujian Province University, Putian 351100, China; 7 Lancaster Medical School, Lancaster University, Lancaster LA1 4YW, UK

**Keywords:** dangerous goods marks, hazard perception, traffic psychology, traffic safety, ERPs

## Abstract

Dangerous goods marks are the most effective means of alerting individuals to the potential dangers associated with the transport of dangerous goods. In order to gain a better understanding of how dangerous goods marks convey risk information, the cognitive processing of dangerous goods marks was examined by measuring event-related potentials (ERPs). We recruited 23 participants, and their ERP data were recorded. We discovered that the dangerous goods marks elicited a larger P200 amplitude and a smaller N300 amplitude, indicating that, compared to other marks, the dangerous goods marks exhibited stronger warning information and drew more attention from the subjects. Simultaneously, dangerous goods marks elicited insufficient emotional arousal in individuals. Therefore, these findings suggest that the designs of dangerous goods marks need to be improved, such as improving the graphic consistency. Changes in ERP patterns can be used to measure the risk perception level of dangerous goods marks, which can be used as an accurate indicator of the effectiveness of warning sign design. In addition, this study provides a theoretical foundation for the cognitive understanding mechanism of dangerous goods marks.

## 1. Introduction

Dangerous goods marks assist individuals in immediately perceiving and comprehending potential risk information when they run into vehicles that are transporting dangerous goods, allowing them to take proper safety precautions. Moreover, dangerous goods marks aid timely rescues by providing the information of the category of the transported goods when an accident occurs, playing a crucial role in mitigating consequences. Frequently, accidents involving the transport of hazardous materials can cause severe threats to human life and the environment. For instance, in July 2019, an oil tanker overturned and caught fire in Nigeria, resulting in 48 deaths and over 90 injuries. In June 2020, a truck transporting liquefied petroleum gas exploded on the Shenhai Expressway in Zhejiang Province, China, killing 20 people and seriously injuring 24 others [1]. Therefore, the pivotal function of dangerous goods marks should not be neglected. In a real situation, individuals need to respond quickly after perceiving the dangerous goods marks. The warning information conveyed by the dangerous goods marks and its degree of emotional arousal in an individual are particularly important because this is an individual’s early cognitive processing activity, which will affect subsequent cognitive processing and decision making. This is related to whether the individual can correctly identify dangerous signals and take corresponding safety precautions. Signs with insufficient warning information and emotional arousal require more cognitive resources, which may lead to an increase in the processing time of individual signs, thereby increasing the possibility of traffic accidents.

Presently, subjective methods such as questionnaires and interviews are typically used by researchers to measure and evaluate the effectiveness of warning signs. The results indicate that people are familiar with the current traffic signs, but their comprehension is limited. For instance, Akple and Sogbe et al. utilized questionnaires, interviews, and observations to examine the understanding of 1205 drivers regarding traffic signs such as caution, supervision, and prohibition. Significant differences were found in the subjects’ understanding of different signs, and the subjects’ ability to correctly identify signs did not imply that they understood the signs. In addition, regression analysis results demonstrated a positive correlation between sign comprehension and traffic compliance [2]. Taamneh et al. administered a questionnaire to 400 driver’s license holders to investigate the relationship between personal characteristics and the familiarity with and comprehension of traffic signs. The familiarity level of the experimental materials was higher than the understanding level [3]. Kirmizioglu et al. investigated 1478 subjects in Turkey, and the results showed that the participants were not familiar with the majority of the signs; only 12 out of 39 signs were correctly identified [4]. Razzak et al. investigated how college students in Bangladeshi understand road safety signs [5].

The AKC model is a synthesis of the research conducted by Laughery and Wogalter on the perception and comprehension of warning indicators. On the basis of the current model for sign comprehension, the AKC model is offered as a three-stage model encompassing attention, knowledge, and compliance [6]. It is also mentioned that design considerations and non-design factors influence the effectiveness of warning signs. The design factors include the logo size, graphics, and colors, whereas the non-design factors consist of the target audience and situational factors. The attention stage encompasses attention acquisition and attention conversion; the knowledge stage encompasses comprehension, memory, and belief; and the compliance stage encompasses compliance intention, motivation, compliance decision, and compliance behavior. In the AKC model, the first step in an individual’s perception of risk is paying attention to warning signs, and only then can the risk be comprehended. The majority of prior studies tested the perception of traffic warning signs by examining questionnaire responses. However, quantitative indicators in questionnaires can only provide limited data support, including the subjective comprehension and familiarity of the signs, as reported by subjects. The physiological processes and cognitive neural mechanisms underlying each phase of a person’s perception, comprehension, and emotional and behavioral responses to warning signs cannot be revealed. In the meantime, the results of self-report methods are influenced by memory and consciousness, which may be biased, particularly for “partially correct” answers [7]. In order to address the aforementioned issues, some researchers are beginning to employ objective ways to examine the readability of signs. For instance, participants were required to observe signs of varying comprehensibility while the differences in EEG signals on the cerebral cortex were recorded [7]. Some researchers also utilized an ERP technique to investigate the symbolism of traffic signs [8].

### 1.1. Electrophysiological Components of Risk Perception

An event-related potential (ERP) is a potential induced by an individual stimulation, with high temporal resolution, which can help evaluate the neural activity in the cognitive process immediately and objectively, and separate and identify a single cognitive process [9]. Dangerous goods marks are supposed to convey strong warning information so that individuals are able to quickly understand. At present, few studies focus on the understanding of dangerous goods marks. Even though there are a few studies that have investigated the understanding of traffic warning signs, it is not clear whether their results are applicable to dangerous goods marks. The waveform amplitude of an ERP in different time windows can provide more detailed objective physiological evidence for the study of the cognitive mechanism of dangerous goods marks [10]. Therefore, this study used behavioral experiments and ERP technology to explore the cognitive process of individuals observing the warning information of dangerous goods marks. The P200 is an early positive wave that appears at about 200 ms after the presentation of a stimulus. This component is related to attention, attention bias, and the novelty of the stimulus, reflecting early rapid spontaneous activity [11,12]. The larger the amplitude, the more attention resources the individual uses [13,14,15]. Studies have shown that individuals have negative attentional bias during cognitive processing; that is, negative stimuli are more likely to attract attention, while a danger signal is a typical stimulus that leads to negative bias [16]. Many previous studies found that the P200 component is related to the early detection of threatening stimuli. For example, negative pictures and positive pictures can also induce P200 components with significant amplitudes, but negative pictures induce greater P200 amplitudes than positive pictures [17,18]. Face pictures with negative emotions also elicited a larger P200 than those with positive emotions [19]. Less attractive face pictures also elicited a larger P200 than highly attractive face pictures [20]. In other types of studies, the recognition of negative stimuli using the P200 component was also confirmed. For example, Qin et al. found that words describing dangerous environmental events induced a larger P200 than words describing safe environmental events [21]. Ma et al. asked subjects to judge the subjective degrees of risk of sign warning words and recorded the ERP amplitudes. They discovered that the participants responded faster to words with subjective high-threat judgments and that the ERP index responded with a P200 with a larger amplitude [22]. Through the evaluation of valence, we found that the dangerous goods mark was a negative stimulus, so this study used the P200 component as a signal for the subject to perceive the sign.

### 1.2. Electrophysiological Components of Emotional Arousal

According to the cognitive model of traffic warning signs, individuals are aroused after noticing them. The N300 was considered to be a sub-component of the N400 in previous studies until Mcpherson and Holcomb et al. found that the N300 component should be separated from the N400 component in the time window and that the distribution of the N300 component on the topographic map was closer to the pre-frontal lobe than the N400 component [23]. Therefore, the N300 is considered to be a separate component. The N300 is a negative component that appears in the frontal and parietal lobes at around 300 ms after stimuli are presented. It is an index that can quickly match visual input and is considered to be an image-specific ERP component that is particularly sensitive to picture stimuli [24,25,26]. In addition, the N300 is an emotion-sensitive component. It is a useful tool to study emotional responses to visual stimuli [27]. This component is related to emotional arousal, reflecting the degree of emotional arousal in an individual due to a stimulus. For example, the oddball paradigm was used to study the warning signs of different shapes. It was found that the N300 amplitude induced by an upright triangle warning sign was larger than that of a circular warning sign, which confirmed that the N300 was more sensitive to image stimuli. The amplitude of the N300 can reflect the emotional arousal due to image [18]. In summary, the N300 component can be used as an indicator of emotional arousal, so this study used the N300 to evaluate the emotional arousal of the subjects.

### 1.3. Study Objectives

In this study, an event-related potential technique was adopted to investigate cognitive processing in the risk perception process of dangerous goods marks, using the P200 and N300 components as indicators. We hypothesized that the dangerous goods mark would convey stronger warning information and would attract more attention resources from the subjects, resulting in a larger P200 amplitude. In addition, we hypothesized that dangerous goods marks would elicit a higher degree of emotional arousal, resulting in a larger N300 amplitude.

## 2. Materials and Methods

### 2.1. Participants

Twenty-five university students majoring in different fields were randomly selected as participants. According to a data inspection, the data of 2 subjects were rejected. The remaining 23 participants ranged in age from 19 to 25 (M ± SD = 21.4 ± 1.5, 11 males and 12 females). Informed consent was acquired from all subjects. All subjects were right-handed native Chinese speakers with no color blindness, normal or corrected vision, and no history of mental illness or intellectual disability.

### 2.2. Experimental Stimuli

**Figure 1:**
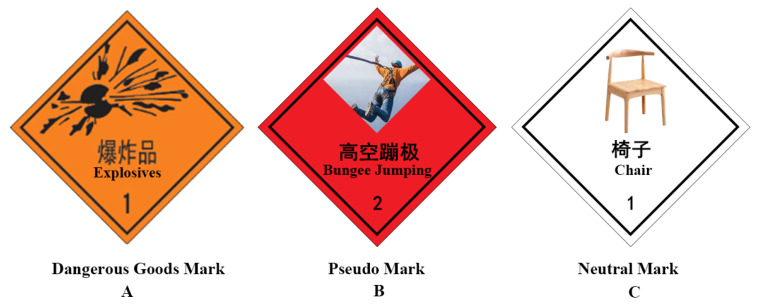
Examples of three types of signs: (**A**) a dangerous goods mark indicating “Beware of explosives”; (**B**) a pseudo-mark indicating “Bungee jumping is risky”; (**C**) a neutral mark.

The dangerous goods marks were selected from *The Vehicle Mark for Road Transportation Dangerous Goods* (Chinese national standard: GB13392, 2005). The content of the pseudo-mark was replaced with pictures of extreme sports such as skydiving, surfing, skiing, rock climbing, bungee jumping, and sailing races [22], and the content of the neutral mark is replaced with pictures of daily items. Neutral marks were only set to maintain the subjects’ focus. Neutral marks were not supposed to arouse positive or negative emotions. Therefore, it was unnecessary to analyze them. All the marks from the three types contained graphics and text. We used a 5-point questionnaire to verify the applicability of the selected experimental materials by examining the valence and arousal scores. In terms of the valence score, 1 represented very negative, 3 represented neutral, and 5 represented very positive. In terms of the arousal score, 1 represented very calm, 3 represented neutral, and 5 represented very excited. A one-way analysis of variance was used to compare the valence and arousal scores of the dangerous goods marks, the pseudo-marks, and the neutral marks. According to the results, significant differences (*F* = 113.652, *ps* < 0.001) were found among the valence scores of the dangerous goods marks (M ± SD = 1.664 ± 0.497), pseudo-marks (M ± SD = 3.810 ± 0.864), and neutral marks (M ± SD = 3.074 ± 0.204). No significant difference (*F* = 26.521, *p* < 0.001) was found between the arousal scores of the dangerous goods marks (M ± SD = 3.871 ± 0.929) and the pseudo-marks (M ± SD = 4.093 ± 0.714). A significant difference was found between arousal scores of the dangerous goods marks and the neutral marks (M ± SD = 2.786 ± 0.673). A significant difference was found between the arousal scores of the pseudo-marks and the neutral marks. Finally, 18 dangerous goods marks, 18 pseudo-marks, and 18 neutral marks were preprocessed using Adobe Illustrator 2018 to standardize their size, color, brightness, and resolution (see Figure 1).

### 2.3. Experimental Procedure

**Figure 2:**
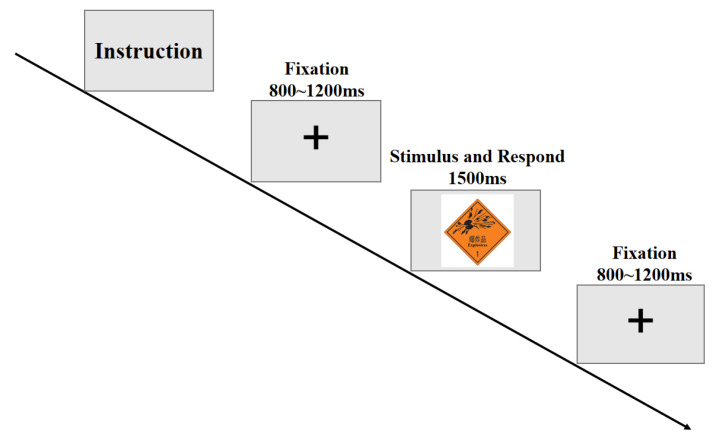
The behavioral experimental procedure.

The behavioral experiment consisted of 2 blocks (72 trials in total). The experimental buffer was set between the groups. Before the formal experiment, the practice trials were carried out. The stimuli were presented in a pseudo-randomized order, and the experimental order of each participant was randomly processed. The experimental materials included dangerous goods marks and pseudo-marks, and the frequencies of presentation were the same. All experimental materials were presented, and the reaction time was recorded using E-prime 2.0 (Psychology Software Tools). The subjects were asked to evaluate the degree of danger after seeing the dangerous goods mark and pseudo-stimuli by pressing on a keyboard (see Figure 2).

**Figure 3:**
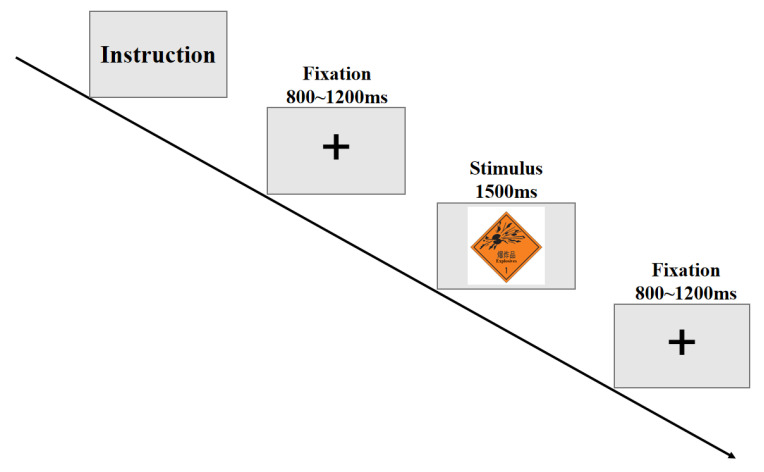
The ERP experimental procedure.

The ERP experiment consisted of 5 blocks (162 trials in total). The stimuli were presented in a pseudo-randomized order, and the order of presentation in each block was different to avoid practice effects. The experimental materials included dangerous goods marks, pseudo-marks, and neutral marks, and the frequencies of the stimuli were the same. The experiment was designed based on a passive viewing task [27] (mark type: dangerous goods mark or pseudo-mark). Subjects were asked to identify and recall the number of neutral marks that had appeared in each trial and report at the end of each block. Before the formal experiment, all participants were fully instructed, and they engaged in a series of practice trials as a mock test. The materials of the practice trials were several other different marks from the same three types. A fixation point was displayed in the center of the screen for a randomized period ranging from 800 to 1200 ms before the stimuli appeared. Dangerous good marks, pseudo-marks, and neutral marks were then presented as stimuli for 1500 ms. In order to avoid action potentials, the subjects were not required to press on the keyboard when the stimuli occurred. Figure 3 depicts the experimental procedure (see Figure 3).

### 2.4. ERP Data Recording

The data were collected using a 64-channel EEG instrument (Brain Product). The experimental procedure was programmed via Presentation (Neurobehavioral Systems) software. The experimental materials were displayed on a 17-inch CRT monitor with a refresh rate of 70 HZ. The electrodes of a 64-channel electrocap were arranged according to the 10-10 international system. The data were recorded using Brain Vision Recorder 2.0. All electrode impedances were maintained below 5 kilo-ohm (kΩ) during the experiment.

### 2.5. ERP Data Analysis

The EEG data were processed offline using Brain Vision Analyzer, Version 2.1 (Brain Products, Gilching, Germany). The EEG activity was sampled at 500 Hz and referenced online to scalp site Cz. The data were re-referenced to the algebraic average of the left and right mastoids. During the experiment, the impedance in all electrodes was ensured to be lower than 5 kΩ. The data were filtered with a 0.1–30 Hz band filter (filter slopes: 24 dB/octave). The horizontal electrooculograms and vertical electrooculograms were collected to identify ocular artifacts in the EEG, which were further corrected with an independent component analysis (ICA). A mean slope average algorithm was selected to detect blink and artifact components. The trials with amplitudes exceeding ±75 µV during stacking were eliminated. ERP segments (200 ms pre-stimulus to 800 ms post-stimulus) were extracted offline. Then, a baseline correction was performed on all segments (200 ms before stimulus onset), and the processed ERP segments were averaged according to the experimental conditions. The data of two subjects were rejected due to excessive (over 30% of the total trials) ocular and physical artifacts during the experiment.

According to previous research [28,29,30] and the grand average map of this study, the amplitude of 170–260 ms at the nine electrodes Fz, F3, F4, Cz, C3, C4, Pz, P3, and P4 was selected to analyze the P200. The amplitude of 300–380 ms at the six electrodes Fz, Cz, F3, F4, C3, and C4 was selected to analyze the N300.

The ERP eigenvalues were analyzed using SPSS 21.0 (IBM Corp., Armonk, NY, USA). The objective of this experiment was to investigate the difference between the individuals’ cognition of the dangerous goods marks and the pseudo-marks, and in terms of neutral marks, participants were only required to count their number of appearances; therefore, the data of the neutral marks were not analyzed. The average amplitude of the P200 for each mark type was analyzed using a two-factor repeated-measures analysis of variance. The mark types (dangerous goods mark and pseudo-mark) and electrodes (9 electrodes) were within-subject variables. The average amplitude of the N300 for each mark type was analyzed using a two-factor repeated-measures analysis of variance. The mark types (dangerous goods mark and pseudo-mark) and electrodes (6 electrodes) were within-subject variables. A Greenhouse–Geisser correction was used to test for sphericity.

## 3. Results

### 3.1. Behavioral Performance

The data from the behavioral experiment included the reaction times of the evaluations of the risk levels of the dangerous goods marks and pseudo-marks. A paired-sample *t*-test was utilized to compare the reaction times of the dangerous goods marks and pseudo-marks. The reaction times of the dangerous goods marks (968.007 ± 113.841 ms) were significantly shorter (*t* = −3.517, *p* < 0.01) than those of the pseudo-marks (1024.809 ± 113.767 ms).

### 3.2. ERP Results

The repeated-measures analysis of variance for the P200 amplitude indicated that the main effect of the mark type was significant (*F*(1,22) = 23.861, *p* < 0.001, ηp^2^ = 0.520), and the P200 amplitude induced by dangerous goods marks was more positive than that induced by the pseudo-marks. The main effect of the electrode position was significant (*F*(8,15) = 3.558, *p* = 0.016 < 0.05, ηp^2^ = 0.655). A further comparison revealed that the P200 amplitude at the left and right positions was significantly more positive than that recorded at the midline position (*ps* < 0.05); the Pz site was significantly more positive than the Cz site (*p* < 0.05); and the Cz site was significantly more positive than the Fz site (*p* < 0.05). In addition, no significant interaction effect between the electrode position and the mark type was observed.

**Figure 4:**
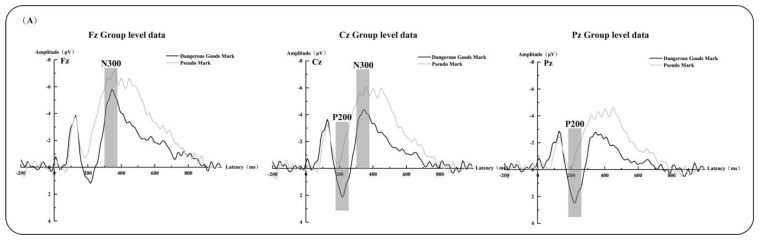

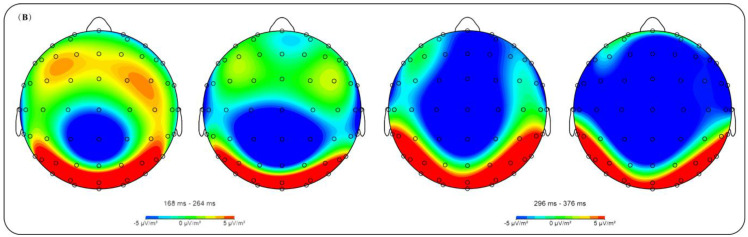
ERP results. Grand average event-related potentials of the P200 and N300 evoked by the standard stimulus at three electrodes in the frontal, central parietal, and parietal regions (FZ, Cz, and Pz): (**A**) grand average event-related potentials evoked by dangerous goods marks and pseudo-marks at three electrodes in the frontal, central parietal, and parietal regions and (**B**) topographic maps of the P200 (170–260 ms) and N300 (300–380 ms) in the dangerous goods mark and pseudo-mark conditions.

The repeated-measures analysis of variance of the N300 amplitude revealed that the main effect of the mark type was significant (*F*(1,22) = 16.343, *p* = 0.001 < 0.005, ηp^2^ = 0.426), and the N300 amplitude evoked by the pseudo-marks was more negative than that elicited by the dangerous goods marks. The main effect of the electrode position was significant (*F*(5,18) = 13.969, *p* < 0.001, ηp^2^ = 0.571). A further comparison revealed that the N300 amplitude at the midline position was significantly more negative than that recorded at the left and right positions (*ps* < 0.05), the Fz site was significantly more positive than the Cz site (*p* < 0.05). In addition, the interaction between the electrode position and the mark type was not significant (see Figure 4).

## 4. Discussion

In this study, the neurophysiological mechanisms of risk perception and the understanding of dangerous goods marks were investigated by analyzing the warning-information-related potentials. Neurophysiological activities in brain regions matched with attention and emotional arousal were revealed by analyzing the P200 (in the central and parietal sites) and N300 components (in the frontal and central sites). The P200 is an indicator of negative attention bias. Compared with pseudo-marks, dangerous goods marks carried more intense warning information, which induced a P200 with a larger amplitude. The N300 is an indicator related to valence and arousal. Compared with the pseudo-marks, an N300 with a smaller amplitude was induced, implying that the emotion evoked by the dangerous goods marks was not intense enough.

### 4.1. Dangerous Goods Marks Might Stimulate an Individual’s Risk Perception

Significant differences were observed in the amplitudes of the ERP components induced by different marks, implying that the design of dangerous goods marks could be evaluated in terms of risk perception and emotional arousal. Under the condition of dangerous goods marks, the amplitude of the P200 component increased significantly during the time period between 170 ms and 260 ms; that is, it attracted more attention resources from individuals, which was consistent with the risk perception hypothesis of the AKC model. Hence, dangerous goods marks could be evaluated from the perspective of risk perception.

The negative attention bias hypothesis states that negative stimuli attract more attention resources than positive stimuli. Ito et al. believe that a stimulus containing risk information is a typical negative stimulus [31]. Eimer et al. asked subjects to judge images of emotional faces and neutral faces, and the results revealed that the negative emotional faces elicited a P200 with a larger amplitude approximately 200 ms after stimulus presentation [32]. Correll et al. recorded the event-related potentials of participants when they were playing shooting games and discovered that armed targets elicited a larger P200 than unarmed targets [17]. Once an accident occurs during the transportation of hazardous materials, it is easy to cause threatening consequences to lives, social security, and the environment, such as a major explosion, intense burning, or poisoning [33]. The aforementioned characteristics could be exhibited by dangerous goods marks for which the picture, text, color, and frame carry stronger risk and warning information. Individuals would spend more attentional resources in the process. In this study, participants received stronger risk information, as indicated by the larger amplitude of the P200 of dangerous goods marks, which was consistent with the findings of previous research. In addition, the results of this study suggest that individuals allocate more cognitive resources to process dangerous goods marks, which is precisely what the designers intended.

### 4.2. Dangerous Goods Marks Elicit a Lower Degree of Emotional Arousal in Individuals

Under the condition of dangerous goods marks, the amplitude of the N300 component increased significantly from 300 to 380 ms but to a lesser extent than in the pseudo-mark condition. The N300 has been shown in previous studies to be a suitable component to distinguish the emotional characteristics of visual stimuli [34]. Larson et al. found that even very simple contexts and free geometric shapes signal emotion, conveying an affect associated with the perception of unpleasantness [35]. In the evaluation of stimulus materials, we proved that the dangerous goods mark has negative valence, so it can be considered to convey emotions related to risk perception to individuals. In other words, the dangerous goods marks can be evaluated from the perspective of emotional arousal.

In this experiment, the N300 component served as an indicator of emotional arousal. Osorio et al. believed that the N300 was associated with emotional responses to visual stimuli in general [36]. The waveform and brain region distribution of the N300 was comparable to that of the N400, reflecting the earliest stages of semantic comprehension [37,38]. Compared with the pseudo-marks, the dangerous goods marks elicited an N300 with a smaller amplitude. In other words, the emotional arousal elicited by dangerous goods marks in individuals was not intense enough. However, there was no significant difference in the subjective arousal scores of the two types of marks, and the subjective results were separated from the objective EEG indicators. This result may be due to the fact that individuals had a longer time to process the marks in the subjective questionnaire (i.e., this might suggest that the subjects required a longer period of time to be fully aroused by dangerous goods marks).

The degree of emotional arousal of individuals in the cognitive process may have different effects on the understanding and decision making in the middle and late stages. From the electrophysiological results of this study, there are defects in the design of the emotional arousal elicited by dangerous goods marks, which may affect an individual’s later cognitive processing and lead to poor decision making in traffic, which is a life-threatening circumstance.

### 4.3. Differences Found in the Main Brain Sites in the Recognition Stage of Dangerous Goods Marks

The P200 and N300 components highlight the perception stage of dangerous goods mark cognition, which was confirmed by the ANOVA results of the mark types and electrode positions. The amplitude of the P200 at the parietal site was significantly larger than those at the frontal and central sites, and the amplitude of the P200 induced by the dangerous goods marks at these sites was significantly larger than that of the pseudo-marks. The amplitude of the N300 at the frontal site was significantly larger than that at the central site, and the amplitude of the N300 induced by the dangerous goods marks at these sites was significantly smaller than that of the pseudo-marks. In other words, during the attention stage of a dangerous goods mark, the P200 amplitude produced by the parietal site was greater than that of the frontal and central sites. A larger N300 amplitude was exhibited at the frontal site than at the central site during the emotional arousal stage. Consistent with the findings of previous research, the results of this experiment demonstrated that the participants’ attention activities for the dangerous goods marks were primarily concentrated at the parietal site, whereas their emotional arousal activities were primarily concentrated at the frontal site [8,28,29,39].

Theoretically, this study offers a novel perspective on the perception of dangerous goods marks. First, this study reveals the behavioral and electrophysiological mechanisms underlying the perception of dangerous goods marks. This is, as far as we are aware, the first study to use the ERP method to investigate the brain mechanism behind the process of recognizing dangerous goods mark indicators. In addition, the results of the ERP experiment indicate that the perceptual processing of dangerous goods marks is separated into stages of attention and emotional arousal, thus partially validating the AKC model [6]. The degree of emotional arousal due to dangerous goods marks is relatively low, indicating that the design of the Chinese national standard for dangerous goods marks is defective.

This study also offers practical implications. Safe transport, especially for hazardous materials, has always been heatedly discussed in the field of transportation management. Warning symbols are one of the most essential safety precautions; hence, the efficiency of dangerous goods marks is of paramount importance. In actual driving conditions and after an accident, the driver and relevant personnel can adopt appropriate safety behaviors based on the warning and information provided by the dangerous goods marks in order to prevent or reduce the occurrence of accidents and prevent further property and personal damage caused by an improper method of rescue. However, based on their subjective impressions following the trial, the majority of participants reported that some dangerous goods marks were difficult to comprehend and provided inadequate notice. In order to improve the safety of road traffic and transport, enhance the efficiency of traffic safety management, and more effectively protect the safety of people’s lives and property, we recommend that the relevant departments further improve the design of dangerous goods marks, such as by making them more vivid so that individuals can more easily perceive and understand the risk information carried by the marks and respond accordingly.

This study also has some shortcomings. Due to the task-independent paradigm, the task of the participants was to count the number of neutral marks. When a non-target stimulus appeared, the understanding and processing of the target stimulus was shorter and did not significantly induce the ERP components of the late cognitive processing. Therefore, the late cognitive processing of the dangerous goods marks was not clear. In future research, the late cognitive process of dangerous goods marks can be further studied, and experimental materials with a stronger sense of presence can be used. In addition, the heterogeneity of the experimental participants can be increased. Specifically, VR and other technologies can be used to simulate real driving and accident situations, and different experimental paradigms can be changed to study the differences in the understanding of dangerous goods marks among different categories of people and whether the use of dangerous goods marks affects the behavior patterns of drivers, passers-by, and rescue workers. In addition, the influence of learning on the understanding of dangerous goods marks can also be studied.

## 5. Conclusions

In this study, event-related potentials were used to examine the cognitive neural processing of individuals as they perceived dangerous goods marks. We observed significant amplitude changes in the P200 and N300 components. In this paper, it is suggested that the amplitude responses of the P200 and N300 ERP components can be used to evaluate an individual’s ability to perceive dangerous goods marks. Specifically, the high-danger-level warning signals carried by dangerous goods marks can activate individuals’ attention for risk and threat information and communicate specific warning information. However, due to design flaws, the individual’s emotional arousal is insufficient, which may affect later understanding, processing, and decision-making behavior. According to the AKC model, warning signs should be able to effectively draw the attention of individuals to potential risks. The results of this study partially validate the AKC model and provide neurophysiological evidence for the design of dangerous goods marks. In addition, this study provides an accurate method for evaluating the effectiveness of dangerous goods marks and may possess good utility for enhancing the safety of transporting dangerous goods.

## Data Availability

The data presented in this study are available on request from the corresponding author.
